# REACTOGENICITY OF HETEROLOGOUS MRNA-BASED COVID-19 VACCINE BOOSTER IN YOUNG ADULTS IN INDONESIA- A SHORT COMMUNICATION.

**DOI:** 10.21010/Ajidv17i2.2

**Published:** 2023-03-29

**Authors:** SURYADINATA Neneng, CHRISTOPHER Paulus Mario, IMANUELLY Michelle, WIJAYA Ratna Sari, CUCUNAWANGSIH Cucunawangsih, LUGITO Nata Pratama Hardjo

**Affiliations:** 1 Faculty of Medicine, Pelita Harapan University, Tangerang, Banten, Indonesia; 2 Department of Microbiology, Faculty of Medicine, Pelita Harapan University, Tangerang, Banten, Indonesia; 3 Department of Internal Medicine, Faculty of Medicine, Pelita Harapan University, Tangerang, Banten, Indonesia

**Keywords:** COVID-19, vaccination, booster, heterologous, mRNA-based vaccine

## Abstract

**Background:**

Heterologous priming with the inactivated SARS-CoV-2 vaccine (CoronaVac) and boosting with mRNA-based COVID-19 vaccine (Moderna or Pfizer) is currently recommended in Indonesia. The reactogenicity data of these heterologous vaccine regimens are not entirely available, particularly in young adults. The present study, therefore, aimed to evaluate the solicited local and systemic reactions in the first seven days post-vaccination either with Moderna or Pfizer vaccine among previous recipients of two doses of CoronaVac.

**Materials and Methods:**

An electronic-based cross-sectional study was conducted among medical students at the Pelita Harapan University, Banten, Indonesia, who received mRNA-based COVID-19 vaccine following two doses of CoronaVac. Samples were collected using a cluster sampling technique. Comparison between groups was performed by Fisher’s exact test.

**Results:**

A total of 72 participants, 23 (32%) of which received the Moderna vaccine and 49 (68%) received the Pfizer vaccine, were included in this study. The median age of participants was 21 (IQR 19-22) years old. The most common local and systemic events for mRNA-based COVID-19 vaccines were injection site pain, fever, headache, fatigue, myalgia, and arthralgia. Solicited local and systemic reactions were reported more frequently in Moderna recipients than Pfizer recipients. Most local and systemic reactions were graded as mild to moderate and did not lead to hospitalization.

**Conclusions:**

The reactogenicity of the heterologous prime-boost with CoronaVac and mRNA-based COVID-19 vaccine booster among young adults is reassuring, and no unexpected concerns were identified.

## Introduction

Vaccination directed toward SARS-CoV-2 has been evaluated as an effective and promising strategy to overcome the COVID-19 pandemic. In Indonesia, the inactivated SARS-CoV-2 vaccine (CoronaVac, Sinovac Life Sciences, China) was [P1]the first vaccine made available and has been widely administered since January 2021. Although the immunogenicity of two doses of CoronaVac vaccine has been shown by a previous study (Zhang *et al*. 2021), the antibody levels predictive for SARS-CoV-2 protection rapidly declined within a few months after vaccination (Jantarabenjakul *et al*. 2022).

The heterologous COVID-19 vaccine booster has been implemented for the general population in Indonesia since January 2022 to overcome the potential of waning immunity. Heterologous booster with mRNA-based COVID-19 vaccine (Moderna and Pfizer) has been recommended for fully vaccinated individuals with CoronaVac due to its capacity to elicit a robust humoral and cellular immune response and provide immunity against SARS-CoV-2 variants (Barros-Martins *et al*. 2021). However, the increased reactogenicity was [P2]frequently reported in heterologous vaccine regimens and was more common in younger age groups (Munro *et al*. 2021; Shaw *et al*. 2021; Clemens *et al*. 2022).[P3] Continued monitoring of reactogenicity after heterologous booster administration in young participants may provide valuable information for the health care practitioner and the public about expected local and systemic reactions following the current available heterologous vaccine regimens.

## Materials and Methods

This cross-sectional study was conducted amongst medical students at the Pelita Harapan University, Indonesia. Ethical approval was given by the Research Ethics Committee, Faculty of [P4]Medicine, Pelita Harapan University (No: 117/K-LKJ/ETIK/III/2022). Written informed consent was obtained from all participants prior to data collection. A total of 72 participants who received two doses of the inactivated COVID-19 vaccine (CoronaVac) were included in this study. Approximately after 6 months, all the fully vaccinated participants received the third dose of the COVID-19 vaccine either with Moderna (mRNA-1273) or Pfizer (BNT162b2).

Safety assessment included monitoring the solicited local and systemic adverse reactions reported by the participants within seven days following the third vaccination. Baseline data on demographics and safety assessment were collected by electronic questionnaire. Perceived severity was evaluated using a modified 4-point Food and Drug Administration Toxicity Grading Scale.[P5] The severity of the adverse reactions was classified into four categories (grade 1, mild; grade 2, moderate; grade 3, severe, and grade 4, potentially life-threatening (Food and Drug Administration, 2007)[P6].

## Results

**Figure 1:**
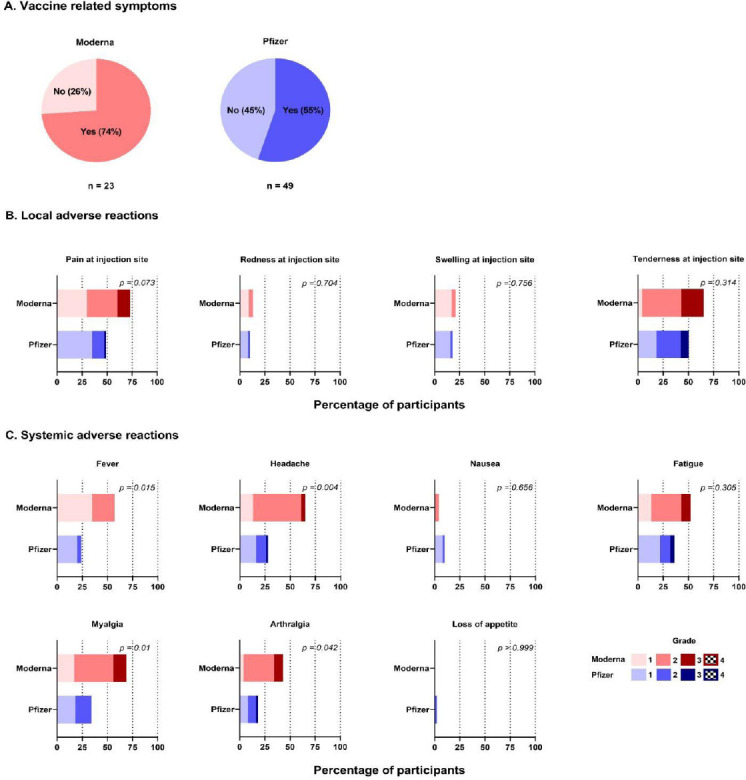
Reactogenicity of mRNA COVID-19 vaccine booster dose within seven days of vaccination.

The median age of participants was 21 years (IQR, 19-22), and 71% of which were females. The majority of participants had no underlying diseases (98%, n = 70). Of the 72 study participants, 23 (32%) individuals previously vaccinated with the CoronaVac series received heterologous regimens with Moderna, whereas 49 (68%) fully vaccinated individuals acquired Pfizer booster. Detailed characteristic of participants is provided in Table 1. After receiving the booster vaccine, participants experiencing at least one of any grades of solicited local and systemic symptoms in the first 7 days were reported in 17 of 23 participants (74%) who received Moderna and in 27 of 49 participants (55%) who received Pfizer (Figure 1A).

**Table 1:** Characteristics of the participants

Variable	All (n=72)
Age (years), median (IQR)	21 (19-22)
Gender, n (%)	
Female	51 (71)
Male	21 (29)
Health status	
Healthy	70 (98)
Immune related diseases	1 (1)
Chronic related diseases	1 (1)
Booster vaccine type	
Moderna	23 (32)
Pfizer	49 (68)

Panel A shows the percentage of Moderna (n = 23) and Pfizer (n = 49) booster recipients that reported any solicited local and systemic reactions in the first seven days after vaccination. Panel B shows the percentage of participants with local adverse reactions (pain, redness, swelling, and tenderness at the injection site), and panel C shows the percentage of participants with systemic adverse reactions (fever, headache, nausea, fatigue, myalgia, arthralgia, and loss of appetite) after booster vaccination. Local and systemic adverse reactions were graded into four categories (grade 1, mild; grade 2, moderate; grade 3, severe, and grade 4, potentially life-threatening). Comparison between groups is performed by Fisher’s exact test.

The most common local adverse reaction was pain at the injection site by 17 (74%) of 23 for Moderna and 24 (49%) of 49 for Pfizer. Tenderness at the injection site was also frequently reported in 15 (65%) of 23 Moderna recipients and 25 (51%) of 49 Pfizer recipients. Most local reactions were graded as mild or moderate. A small number of participants had severe local reactions and its more commonly found in participants who received the Moderna vaccine (**Figure 1B**). As shown in **Figure 1C**, a higher percentage of systemic adverse reactions (fever, headache, myalgia, and arthralgia) were reported from Moderna recipients than from Pfizer recipients. Further analysis revealed there was a statistically significant association between solicited adverse reactions (fever, headache, myalgia, and arthralgia) with vaccine type (p<0.05). Most of the systemic reactions were mild to moderate and did not lead to hospitalization.

## Discussion

In the current study, heterologous vaccine regimens with CoronaVac and mRNA-based COVID-19 vaccine were found to be well-tolerated among young individuals (19 to 22 years of age). There were no participants who reported grade 4 local and systemic reactions that required hospitalization or medical attention. The most frequently reported solicited local and systemic reactions in the first seven days after mRNA-based COVID-19 vaccination was pain at the injection site, fever, fatigue, headache, myalgia, and arthralgia. This result was generally consistent with previous studies of mRNA-based COVID-19 vaccine safety (Polack *et al*. 2020; Baden *et al*. 2021; Chaptin-Bardales *et al*. 2021). The higher reactogenicity was found in Moderna booster recipients than in those who received the Pfizer vaccine. The findings of this study are in line with the findings reported in the COV-BOOST study, in which greater reactogenicity was identified in Moderna booster recipients than in Pfizer recipients (Munro *et al*. 2021).

## Conclusion

Although the local and systemic reactions of the COVID-19 vaccine were common among the young age group, our study showed that the administration of mRNA-based COVID-19 vaccine in young adults vaccinated with CoronaVac was well-tolerated. Identifying expected local and systemic reactions after mRNA-based COVID-19 vaccination was important to alleviate the participants’ anxiety regarding the vaccination experience and increase the acceptance of the current heterologous COVID-19 vaccine regimens.

### Competing Interests

The authors have declared that no competing interest exists.

**List of abbreviations:**
COVID-19: Coronavirus Disease 2019.,
mRNA: Messenger RNA.,
SARS-CoV-2: Severe Acute Respiratory Syndrome Coronavirus 2.
